# An Updating System for the Gridded Population Database of China Based on Remote Sensing, GIS and Spatial Database Technologies

**DOI:** 10.3390/s90201128

**Published:** 2009-02-20

**Authors:** Xiaohuan Yang, Yaohuan Huang, Pinliang Dong, Dong Jiang, Honghui Liu

**Affiliations:** 1 State Key Lab of Resources and Environmental Information System, Institute of Geographical Sciences & Natural Resources Research, Chinese Academy of Sciences (CAS) / Beijing 100101, P.R. China. E-Mails: huangyh@lreis.ac.cn; jiangd@igsnrr.ac.cn; liuhh@igsnrr.ac.cn; 2 Graduate School of the Chinese Academy of Sciences / Beijing 100049, P.R. China; 3 Department of Geography, University of North Texas / 1155 Union Circle #305279, Denton, TX 76203, USA. E-Mail: pdong@unt.edu

**Keywords:** Gridded population, Population database, Remote sensing, GIS, Land use

## Abstract

The spatial distribution of population is closely related to land use and land cover (LULC) patterns on both regional and global scales. Population can be redistributed onto geo-referenced square grids according to this relation. In the past decades, various approaches to monitoring LULC using remote sensing and Geographic Information Systems (GIS) have been developed, which makes it possible for efficient updating of geo-referenced population data. A Spatial Population Updating System (SPUS) is developed for updating the gridded population database of China based on remote sensing, GIS and spatial database technologies, with a spatial resolution of 1 km by 1 km. The SPUS can process standard Moderate Resolution Imaging Spectroradiometer (MODIS L1B) data integrated with a Pattern Decomposition Method (PDM) and an LULC-Conversion Model to obtain patterns of land use and land cover, and provide input parameters for a Population Spatialization Model (PSM). The PSM embedded in SPUS is used for generating 1 km by 1 km gridded population data in each population distribution region based on natural and socio-economic variables. Validation results from finer township-level census data of Yishui County suggest that the gridded population database produced by the SPUS is reliable.

## 1. Introduction

Demographic data is one of the most direct indexes of the influence of human activities on the planet Earth. Application areas of demographic information include ecosystem assessment, global environmental changes, public health, and regional sustainable development studies [1-7]. Traditionally, census datasets of administrative or statistical reporting units could not meet the needs of these applications because of (1) the low spatial resolution of the datasets, and (2) the long time interval between census years. A new concept of “population spatialization” was presented in a workshop on Global Demography in 1994 for redistributing population onto geo-referenced grids instead of political or administrative units [8-9]. Since Tobler *et al.* released the first version of Gridded Population of the World (GPW) in 1995 [8], great progress has been made by many scientists and organizations in creating population databases. One example is the Gridded Population of the World (GPW) developed by the Center for International Earth Science Network (CIESIN) at Columbia University to depict the distribution of human population across the globe [10-14]. The latest developments were Gridded Population of the World, version 3 (GPWv3) and the Global Rural-Urban Mapping Project (GRUMP). GRUMP builds on GPWv3 by incorporating urban and rural information, allowing new insights into urban population distribution and the global extents of human settlements [15]. Another example is the LandScan Global Population Dataset developed by the Oak Ridge National Laboratory (ORNL) as a worldwide population database for ambient population at 30″ by 30″ resolution [16, 17]. Census counts (at sub-national level) were apportioned to each grid cell based on likelihood coefficients, which are based on proximity to roads, slopes, land cover types, nighttime lights, and other information.

In 2002, the Data Center for Resources and Environmental Sciences (RESDC) of the Chinese Academy of Sciences (CAS) developed the China Gridded Population Datasets of 1995 and 2000 for residential population with a spatial resolution of 1 km by 1 km [18]. By the end of the year 2006, the China Gridded Population Datasets of 2005 was also produced by RESDC. This paper focuses on the development of the Spatial Population Updating System (SPUS) for automated updating of annual gridded population databases based on LULC data derived from the Moderate Resolution Imaging Spectroradiometer (MODIS) images acquired by NASA's Earth Observing System (L1B data of bands 1-7 with 500 m spatial resolution). Validation results and future directions of improvement are also discussed.

## 2. Data and Methodology

### 2.1. Data Sources

The data of the research include the Chinese census data, land use data and ancillary data listed in Table 1. According to the differences in data sources and data types, the criterion and precision also vary. Data preprocessing has been conducted for all data layers, including satellite image correction, projection transformation, and attribution data standardization.

### 2.2. Methodology for Land-use/Land-cover (LULC) Data Updating

The Chinese land use types were used as primary indices in the spatial population model of this paper. The Pattern Decomposition Method (PDM) is applied to MODIS data to obtain the land use/land cover patterns. The vegetation, water and soil coefficients are extracted by PDM for each MODIS image pixel to create a LULC-Conversion Model (LULC-CM). The MODIS images are classified into different land use types based on the relationship between the spectral coefficients and the land-use structure. To eliminate the noise effects to MODIS bands, e.g. cloud effects, we merged the clearest images between July and September which corresponds to the period when vegetation flourished. Thus it was easier to classify the land cover of built-ups and bare soil areas caused by crop harvesting, which is sufficient for modeling spatial distribution of population in annual increments.

#### (1) Pattern Decomposition Method (PDM)

Spectral response patterns for each pixel of an image can be decomposed into three components using three standard spectral shape patterns determined from the image data [19, 20]. Zhang has used the *PDM* to detect land cover in Miyun District in Beijing of China, and found that there are good correlations between the bands of VIS and LULC type and area [21]. The LULC information can be represented by three standard spectral patterns: vegetation, water and soil, transferred from the 7 VIS bands of MODIS data.

First of all, standard spectrum patterns are extracted from the MODIS L1B data. Surface albedos of the MODIS L1B data within the specified experimental area are normalized to avoid the disturbance of absolute spectrum values. The normalization albedo can be calculated using:

(1) $$B_{i}=(\frac{A_{i}}{\sum_{j=1}^{7}A_{j}}).$$

where *B_i_* is the normalized albedo value of band i; *A_i_* is the original surface albedo value of band i; *A_j_* is the original surface albedo value of band j; and j is the index of image bands. After choosing the sample pixels of pure water, soil and vegetation in the test area, a 3 × 7 matrix P is obtained by averaging the *B_i_* values of the 7 bands. The *B_i_* values represent the spectrum pattern of water, vegetation and soil in MODIS band 1 to 7. Taking Shandong Province as an example, the standard spectrum patterns are shown in Table 2.

Secondly, pixel spectrum decomposition is conducted based on the standard spectrum patterns. The albedo of each pixel is expressed as a linear combination of the reflectance of each LULC unit:

(2) $$A_{i}=C_{w}P_{\text{iw}}+C_{v}P_{\text{iv}}+C_{s}P_{\text{is}}+R$$

where *A_i_* is the surface albedo of the pixel at band i; *P_iw_*, *P_iv_*, and *P_is_* are the standard spectrum patterns of water, vegetation and soil; and *C_w_, C_v_*, and *C_s_* represent decomposition coefficients (positive numbers). According to the PDM principle, proportions of the three types of land use in each pixel can be obtained using:

(3) $$r_{k}=\frac{C_{k}}{S_{k}}/\left(\frac{C_{w}}{S_{w}}+\frac{C_{v}}{S_{v}}+\frac{C_{s}}{S_{s}}\right),k=w,v,s.$$

where *r_w_, r_v_*, and *r_s_* represent the three matrices for water, vegetation and soil proportions in a pixel of 500 m × 500 m.

#### (2) LULC-Conversion Model (LULC-CM)

The results obtained from Equation (3) cannot be converted to land use types directly. However, the combination of *r_w_, r_v_*, and *r_s_* can be associated with land use types. This paper uses the decomposition results and LULC-CM to derive land use types. The LULC-CM is created using the following equation:

(4) $$C_{1}\cdot X_{1}=Y\to Y=[C_{2}C_{3}]\cdot {\left[r_{w}r_{v}r_{s}1\right]}^{\prime }.$$

where *C_1_, C_2_, C_3_, X_1_,* and *Y* are matrices, and *X_1_* = *[x1, x2, x3, x4, x5, x6]*, representing different percentages of cropland, forest, grassland, urban residential, rural residential, and water area in each pixel; *C_1_, C_2_,* and *C_3_* are modulus of the model and the constant part; *r_w_, r_v_, r_s_* are obtained from Equation (3).

### 2.3. Method of Population Spatialization

**Figure 1.**
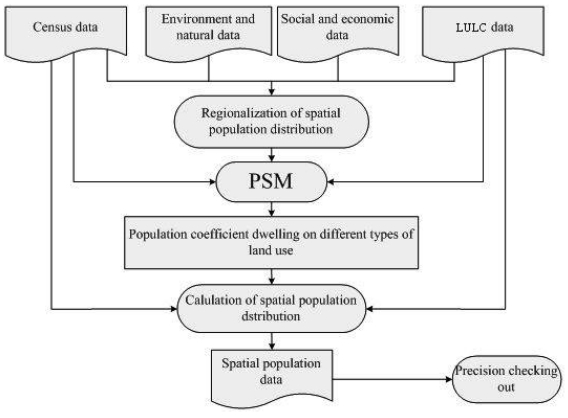
Flowchart for producing Gridded Population Dataset of China.

This paper adopts the method of population spatialization model based on the relationship between demographical data and land use types, and redistributes population onto 1 km by 1 km grids [18]. The Data Center for Resources and Environmental Sciences of the Chinese Academy of Sciences (RESDC, CAS) has applied this method to build the 2000 gridded population database of China. The general steps for redistributing population are shown in Figure 1.

#### (1) Regionalization of Spatial Population Distribution

China is a large country with different population density and land use patterns from the west to the east. According the Fifth Census of China in 2000, the average population densities of the eastern, central, and western China are 452.3, 262.2 and 51.4 (persons/km^2^), respectively. To obtain more reliable results, we constructed a three-dimensional feature space based on the provincial population density core and regionalization index to divide the whole country into 8 regions using the minimum distance rule [21]. A population model was established for each region using the following steps: 1) Calculation of spatial population characteristic index. Based on population density, economic development, land use structure, transportation network, and river density at county level, Equation 5 is used to calculate the spatial population characteristic index of the model.

(5) $$I_{p}=(P+\text{GDP})/S/(1-I_{c})/(1-I_{\text{rd}})/(1-I_{\text{rl}})/(i-I_{r})$$

where *I_p_* is the spatial population characteristic index; *P* is the total population; *GDP* is the gross domestic product; *S* is the total land area; *I_c_* is the index of cultivation density ; *I_rd_* is the road density; *I_rl_* is the railway density; and *I_r_* is the residential density. 2) Determining the center of provincial population distribution. Based on the characteristics of a provincial population distribution index (z) and the spatial distribution of population centers (x, y), a three-dimensional space to calculate inter-provincial population distribution distance (d) is constructed, and the minimum distance method is used to divide the first level into eight population regions. 3) Secondary population regions are divided by county-level terrain parameters and population density. Due to the limitations of space, the details can be found in our previous research [18].

#### (2) Population Spatialization Model (PSM)

Quite a few types of methods for distributing census data have been proposed in recent years. The LandScan Global Population Dataset was produced according to a linear relationship between census counts (at sub-national level) and distribution of roads, slope, land cover, nighttime lights [16, 17]. Mennis and Hultgren presented an “intelligent” dasymetric mapping technique (IDM), which used the ratio of class densities to redistribute population to sub-source zone areas [23].

A population spatialization model is built to redistribute population of a county into different types of land uses:

(6) $$P_{i}=\sum_{j=1}^{n_{f}}{\text{a}}_{j}x_{\text{fj}}+B_{i}$$

where *P_i_* stands for the total population of the i-th county, *a_j_* is the population density of the j-th land use type, *x_fj_* is the total area of the j-th land use type in f-th section (km^2^), and *n_f_* is the number of land use types in the current section. According to the rule of “no residential area, no population”, the intercept *B_i_* is set to zero.

To ensure that predicted population equals to the census statistical results within each administrative unit, the population density of each land use type should be adjusted by the ratio of the predicted population (P_i_) and the census count (P_i_^0^). The initial coefficient *a*_j_ of the adjustment is defined as:

(7) $$a_{\text{ij}}=\frac{P_{i}^{0}}{P_{i}}\times a_{j}$$

where *a_ij_* is the modified population density for the *j*-th land use type within the *i*-th administrative unit; *P_i_* and *P_i_^0^* are the predicted population and census count of the i-th administrative unit respectively.

After the above-mentioned steps, population can be estimated from cell to cell. To create spatialized population data of China, the population of each cell is calculated by linking population density coefficient *a_ij_* to land use grid using the following equation:

(8) $$P_{i}=\sum_{j=1}^{n}a_{\text{ij}}x_{j}.$$

## 3. SPUS System Design and Development

SPUS is a software system combining PDM, LULC-CM, and PSM. Functionalities of SPUS include generation, analysis, visualization, and management of gridded population databases. The system is developed in Microsoft Windows environment and is easy to use. The input to the system includes census data, remotely sensed (such as MODIS) data, and other ancillary data.

### 3.1. Flowchart for Data Processing

**Figure 2.**
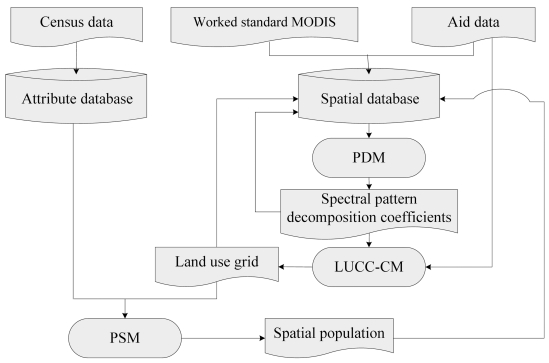
Data process flow of SPUS.

Figure 2 shows the data processing flowchart of SPUS. The input data includes statistical population data at county level, MODIS/Terra Surface Reflectance 8-Day L3 Global 500 m SIN Grid, and ancillary data such as administrative boundary maps. All data layers are stored in the attribute and spatial databases.

**Figure 3.**
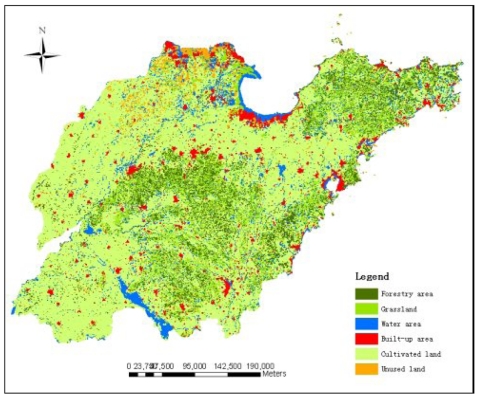
Land use data of Shandong Province, 2002.

As an example of input data layers for SPUS, Figure 3 shows the land use data of Shandong Province in 2002.

### 3.2. System Function Development

**Figure 4.**
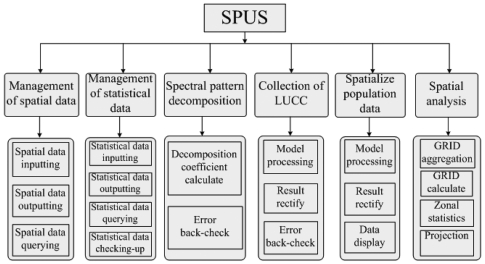
Framework of function modules.

Figure 4 shows the schematic representation of system function modules.

#### (1) Management Module of Spatial Data

The module is a spatial database tool that manages all spatial data based on ArcSDE. It can be used to import, export and query spatial data (including raster and vector databases).

#### (2) Management Module of Statistical Data

The module manages all kinds of statistical data including population, socio-economic data and ancillary attribution data using Oracle 9i. The functions include data input, storage, output with common formats such as Excel, XML, MDB, DBF, data checking, quality control, and data querying.

#### (3) Spectral Pattern Decomposition Module

The module executes the PDM to conduct processing of standard MODIS data to generate ASCII files of coefficients using IDL in the .Net environment. ArcEngine is used to convert ASCII files to GRID format. The module is a mixture of .Net, IDL and ArcEngine.

#### (4) LULC-CM Module

This is a functional module that executes LULC-CM. The module includes three steps: 1) Train the sample areas selected according to the regionalization to get the modulus which can be considered repository of SPUS as the basic information of LULC. 2) Apply the LULC-CM to convert the result of PDM to six different land use types defined as cropland, forest, grassland, city, rural residential area and water in a pixel of 500 by 500 meters. 3) Check and import LULC data into the spatial database. Considering the advantage of IDL in scientific computation, LULC-CM was developed by IDL. The IDL program generates ASCII files with the percentages of six LULC types in each pixel, and then uses ArcEngine to create LULC grids for spatial analysis.

#### (5) Population Data Spatialization Module

The module is the core of SPUS. It executes the PSM to redistribute the statistical population data on grids by combining the LULC grid, province vector data, county vector data and other ancillary data. It is a GIS system developed with ArcEngine consisting of three major functions.

- Data display. The main interface that can add and display spatial data and provide functions of querying, panning, zooming in, zooming out, selecting and saving.
- Model processing. To create spatial population grids based on PSM.
- Results verification. To adjust the primary results according to total population control within county level units to generate the final result of gridded spatial population dataset.

#### (6) Spatial Analysis Module

SPUS provides some common functions of GIS spatial analysis such as grid calculation, projection transformation, aggregation, buffering, overlaying (union, intersect, erase), zonal and neighborhood analysis. Users can combine these functions easily to obtain new spatial indices such as regional total population, population growth rate, and degree of population aggregation.

## 4. Results

**Figure 5.**
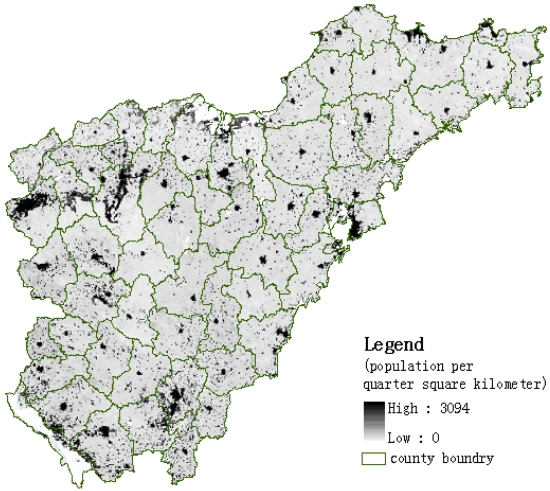
500 m by 500 m gridded population data of Shandong Peninsula in 2002.

The most important functionality of the SPUS system is to update gridded population data. Figure 5 shows the gridded population data of Shandong Province of China in 2002 based on MODIS data. The spatial resolution of grid is 500 m by 500 m, and the maximum value of population in 0.25 sq km grid in Shandong Province is 3,094 persons. There are some high-value areas on the image within county-level administrative units showing the urban areas with higher population density.

Verification of redistributed China population data is time-consuming mainly because of the difficulty in establishing a suitable reference database for comparison purposes. It is difficult to get actual census counts for 1 km × 1 km grids. However, census data of sub-county units, which is township level in China, could be obtained in some provinces. A substitute approach has been designed for verification based on these population data. The following steps were carried out for towns with census data: (1) Create vector boundary maps of towns with census data; (2) Overlay gridded population data with boundary maps; (3) Accumulate population of all cells within these towns; and (4) Compare population estimations with census data. We have collected census data of 19 towns in Yishui County, Shandong Province. Verifications have been conducted using 2002 statistical data.

Table 2 shows that the relative errors between predicted population and actual census counts vary from 0.51% to 25.63%, with eight towns lower than 10%, eight higher than 20%, and the remaining three between 10%-20%. The accuracies are acceptable for many applications at the county, province, or national levels.

## 5. Conclusions

This paper describes the design and implementation of the Spatial Population Updating System (SPUS) integrating GIS, remote sensing, spatial database, and statistical methods. The system combines several modules seamlessly for efficient generation, analysis, visualization, and management of spatial population datasets. Preliminary verification of 2002 gridded population datasets using township level census data suggests that the gridded population datasets can be used for many applications at a regional or national scale. Accuracies of gridded population data derived from currently available global remote sensing data are inadequate for modeling population distribution and detecting changes in the spatial distribution of population on annual increments. Further research in two major directions may be important. The first is to improve model accuracy with more factors related to the spatial distribution of population (especially in urban areas), and validate population models with more accurate census counts at finer resolutions. The other is to extend the SPUS functions for more real applications, such as adding new population indices to help users build spatial population databases.

## Figures and Tables

**Table 1.** List of the Data Sources.

**Data type**	**Time**	**Data sources**	**Scale or resolution**
Census data	1995∼2002	State Bureau of Statistics of China	County level
MODIS L1B	2002	EOS website	500 m
Statistical social and economic data	2000	Chinese Statistic Yearbooks	County level
Land use	2000	Resources and Environmental Scientific Data Center (RESDC), Chinese Academy of Sciences (CAS)	1: 100,000
DEM data	2000	State Bureau of Surveying and Mapping of China	1: 250,000
Boundary of counties	2000	RESDC	1: 100,000
Residential map	2000	State Bureau of Surveying and Mapping of China	1: 250,000
Statistic population of Yishui County, Shandong Province	2002	Statistic Yearbook of Yishui County	Township level

**Table 2.** Standard spectrum patterns in Shandong Province.

	**Band1**	**Band2**	**Band3**	**Band4**	**Band5**	**Band6**	**Band7**
Water pattern P_w_	0.30589	0.23765	0.17209	0.11474	0.07647	0.05731	0.03585
Vegetation pattern P_v_	0.08983	0.08372	0.06711	0.24593	0.24880	0.17251	0.09210
Soil pattern P_s_	0.09182	0.08918	0.09733	0.17880	0.20528	0.19181	0.14579

**Table 2.** Results validation with census data of 19 sample towns in Yishui County

**No.**	**Town name**	**Population (statistical)**	**Population (gridded)**	**Error**	**Relative error (%)**
1	Quan Li	37,256	46,805	9,550	25.63
2	Sha Gou	68,566	83,018	14,451	21.08
3	Ma Zhan	67,456	54,435	-13,021	-19.30
4	Fu Guanzhuang	46,085	57,410	11,325	24.57
5	Yang Zhuang	63,408	61,389	-2,019	-3.18
6	Zhe Ge	75,325	90,975	15,650	20.78
7	Gao Qiao	60,146	46,109	-14,037	-23.34
8	Quan Zhuang	34,293	34,118	-175	-0.51
9	Yi Shui	175,136	152,313	-22,823	-13.03
10	Dao Tuo	36,831	36,831	-2,529	-6.43
11	Gao Zhuang	51,507	50,914	-593	-1.15
12	Long Jiaquan	61,231	47,691	-13,540	-22.11
13	Chui Jiayu	33,380	39,101	5,721	17.14
14	Huang Shanpu	50,252	37,842	-12,410	-24.70
15	Xu Jiahu	81,431	76,713	-4,718	-5.79
16	Yuan Dongtou	29,191	36,185	6,994	23.96
17	Si Shilipu	66,312	61,245	-5,067	-7.64
18	Yao Dianzi	44,766	40,533	-4,233	-3.52
19	Xia Wei	54,898	52,967	-1,931	-2.93
